# Hierarchical Ti-MOF Microflowers for Synchronous Removal and Fluorescent Detection of Aluminum Ions

**DOI:** 10.3390/bios12110935

**Published:** 2022-10-27

**Authors:** Jianguo Zhou, Jieyao Song, Guangqiang Ma, Yongjian Li, Yanan Wei, Fei Liu, Hongjian Zhou

**Affiliations:** 1 Institutes of Physical Science and Information Technology, Anhui University, Hefei 230601, China; 2 Key Laboratory of Materials Physics, Centre for Environmental and Energy Nanomaterials, Anhui Key Laboratory of Nanomaterials and Nanotechnology, Institute of Solid State Physics, HFIPS, Chinese Academy of Sciences, Hefei 230031, China; 3 Department of Materials Science and Engineering, University of Science and Technology of China, Hefei 230026, China; 4 School of Mechanical and Electrical Engineering, Xi’an University of Architecture and Technology, Xi’an 710055, China

**Keywords:** luminescence, metal-organic frameworks, hierarchical nanostructures, fluorescent sensors, metal ions

## Abstract

Bifunctional luminescence metal-organic frameworks with unique nanostructures have drawn ongoing attention for simultaneous determination and elimination of metal ions in the aqueous environment, but still remain a great challenge. In this work, three-dimensional hierarchical titanium metal-organic framework (Ti-MOF) microflowers were developed by a secondary hydrothermal method for not only highly sensitive and selective detection of Al(III), but also simultaneously efficient decontamination. The resulting Ti-MOF microflowers with a diameter of 5–6 μm consisted of nanorods with a diameter of ∼200 nm and a length of 1–2 μm, which provide abundant, surface active sites for determination and elimination of Al(III) ions. Because of their substantial specific surface area and superior fluorescence characteristics, Ti-MOF microflowers are used as fluorescence probes for quantitative determination of Al(III) in the aqueous environment. Importantly, the specific FL enhancement by Al(III) via a chelation-enhanced fluorescence mechanism can be utilized for selective and quantitative determination of Al(III). The Al(III) detection has a linear range of 0.4–15 µM and a detection limit as low as 75 nM. By introducing ascorbic acid, interference of Fe(III) can be avoided to achieve selective detection of Al(III) under various co-existing cations. It is noteworthy that the Ti-MOF microflowers exhibit excellent adsorption capacity for Al(III) with a high adsorption capacity of 25.85 mg g^−1^. The rapid adsorption rate is consistent with a pseudo-second order kinetic model. Ti-MOF is a promising contender as an adsorbent and a fluorescent chemical sensor for simultaneous determination and elimination of Al(III) due to its exceptional water stability, high porosity, and intense luminescence.

## 1. Introduction

Aluminum is a substance that is commonly present in the environment and in human activities, is thought to hinder the growth of plants, and is linked to diseases such as Alzheimer’s and Parkinson’s diseases [1]. The World Health Organization (WHO) recommends that the maximum aluminum content in drinking water should be limited to 7.4 µM, and the daily consumption of aluminum for humans should be between 3 and 10 mg [2]. Measurement and purification of Al(III) is essential for environmental monitoring and preserving human health, since aluminum often exists in the body and environment as the Al(III) ion [3]. Consequently, significant efforts are being undertaken to develop novel approaches for the selective detection and removal of Al(III) because of its pervasive contamination and high toxicity. For sensitive detection of Al(III) pollution in the aqueous environment, a variety of atomic absorption or emission spectrometry methods, inductively coupled plasma mass spectrometry (ICP-MS), and fluorescence methods, have been utilized [4,5]. Due to its ease of use, cheap cost, convenience, sensitivity, real-time monitoring, and naked-eye visibility, fluorescence based chemosensors have been identified as promising tools for detecting Al(III). Nevertheless, sensitive and selective detection of Al(III) is still tedious, with several disadvantages due to its lack of spectroscopic features, sluggish coordination, and a great hydration capacity. 

Toxic metals must be removed from polluted water. Many methods have been developed recently to remove harmful metals from diverse substances, including ion displacement, membrane filtration, redox co-precipitation, chemical deposition, absorption methods, and solid-phase extraction [6,7]. Among these technologies, adsorption is believed to be the most cost effective method to remove Al(III) from contaminated water because of its simplicity of use and the availability of a variety of adsorbents, such as *Streptomyces rimosus* biomass, Fe_3_O_4_/TEOS/AMEO/GA, activated carbon, and polyacrylonitrile beads [8]. Unfortunately, most of the previous studies either concentrated on functional adsorption materials to improve selectivity and sensitivity towards metal ion detection or centered on absorbing materials to boost their uptake capacity for metal ions, which seriously hindered their practical applications. Developing a novel bifunctional material that can both identify and eliminate metal ions from environmental samples is of ongoing interest, and would significantly increase the fluorescence signal due to effective adsorption. Qian et al. reported a covalently grafted naphthalimide derivative of 2,6-bis(aminomethyl)pyridine on the surface of silica particles to create a bifunctional fluorescence sensor for the simultaneous detection and separation of trace Hg^2+^ in polluted water samples [9]. Manos et al. developed a microporous metal-organic framework (H_16_[Zr_6_O_16_(H_2_PATP)_4_]Cl_8_·xH_2_O) that has an exceptional capacity to quickly collect and selectively detect hexavalent chromium in aqueous solution [10]. Our group also reported the selective detection and simple removal of arsenate from contaminated water using amino-functionalized iron-based metal organic framework (NH_2_-MIL-88(Fe)) nanooctahedra [11]. However, to the best of our knowledge, such bifunctional materials that are capable of detecting and removing Al(III) from environmental samples simultaneously have not been reported to date. 

To meet this demand, we attempted development of a bifunctional material with the following characteristics: suitable recognition sites to provide specific receptor-target interactions for luminescence signal responses, strong metal chelating groups with high affinity toward specific target metal ions, ordered and high-density accessible binding sites, exceptional water and chemical stability to facilitate multiple uses, and high efficiency in converting light into electrical energy. To fulfill this goal, luminescence metal-organic framework (LMOF) materials are ideal candidates that offer a special scaffold and tailored functionality with luminescence in integrating two functions of sensing and adsorption into a single material, in contrast to conventional sensing and remediation materials [12,13,14,15]. Numerous experiments using MOF-based sensors have recently been published to detect Al(III) [16,17]. For instance, Zhan, et al. developed a water-resistant terbium-MOF sensor for the accurate, precise, and recyclable detection of Al (III) [17]. To ratiometrically detect Al^3+^ ions in an aqueous solution, Zheng et al. created a new zirconium-based MOF composite material called UiO-(OH)_2_@RhB [18]. Design of an MOF-based sensor for simultaneous detection and removal of Al(III) in an aquatic environment, however, remains a formidable issue. 

Adsorption efficiency can be significantly impacted by changing the geometry of adsorbents. This suggests that in order to reduce the diffusion barrier and increase adsorption efficiency, research on the design of adsorbents at the geometrical level should be pursued. The integration of nano-building blocks with the proper order and flexibility to create hierarchical nanostructures has proven to be a successful strategy for improving adsorption performance [19]. Hierarchical nanostructures have been successfully created using a wide range of materials, including metals, metal oxides and sulfides, carbons, and certain organic polymers [20]. Metal-organic hierarchical nanostructures, however, are seldom explored. Even though MOF sensing applications have been of interest, it is often challenging to create a free-standing, three-dimensional (3D) hierarchical micro-/nano-structured MOF that has nanoscale thickness and microsized lateral dimensions. A collection of hierarchical metal-organic framework nanostructures has recently been published by Wang et al. [21,22]. These substances differ from their pure organic or inorganic equivalents in terms of functions, and they may find use in a variety of industries. Based on these advantages, we assumed that the integration of 3D hierarchical micro-/nano-structure and bifunctional LMOF would be highly desired and could improve fluorescence sensing and adsorption performance. 

**Scheme 1:**
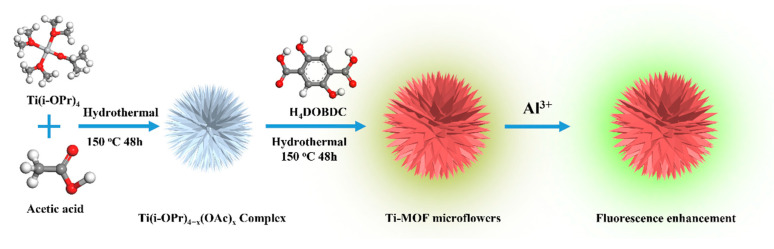
Schematic illustration for manufacturing hierarchical Ti-MOF microflowers and Al(III) ions luminous sensing.

Following these criteria, we developed a novel approach for building three-dimensional (3D) hierarchical titanium-based MOF (Ti-MOF) microflowers by a secondary hydrothermal process for potential use dual-functional fluorescence sensors and adsorbents (Scheme 1). Using a variety of methods, including scanning electron microscopy (SEM), transmission electron microscopy (TEM), X-ray diffractometer (XRD), and fluorescence spectroscopy, the structural features of the obtained product were carefully examined. The sensing characteristics of the developed materials toward Al(III) were investigated, including sensitivity and selectivity in the presence of various interfering ions, to illustrate possible bifunctional applications. Meanwhile, the concomitant adsorption performance of Al(III) onto hierarchical Ti-MOF microflowers was carefully evaluated. Using X-ray photoelectron spectroscopy (XPS) and Fourier transform infrared (FT-IR) spectroscopic studies, the fundamental sensing and adsorption mechanism of hierarchical Ti-MOF microflowers towards Al(III) was investigated. 

## 2. Experimental Section

### 2.1. Synthesis of Hierarchical Ti-MOF Microflowers

According to a previously published approach, with certain modifications, hierarchical Ti-MOFs microflowers were created by a secondary hydrothermal process [23,24]. First, 178 mg Ti(i-OPr)_4_ was dissolved in 10 mL acetic acid with stirring until a milk white emulsion formed. Then, after being transferred, the mixture was heated at 150 °C for 48 h in a 30 mL Teflon-lined stainless-steel autoclave. The autoclave was allowed to cool naturally to ambient temperature before the solution was centrifuged for six minutes at 8000 rpm to remove the supernatant solution, and washed with deionized water three times. Secondly, the achieved white precipitate was re-dispersed in 10 mL of deionized water, and 124 mg H_4_DOBDC was injected to the above solution with stirring. A 30 mL Teflon-lined stainless-steel autoclave was used to transfer the mixture, which was heated at 150 °C for 48 h. Finally, the dark red crystals were centrifuged at 8000 rpm for 6 min and rinsed with deionized water and ethanol three times. 

To confirm that the synthesis of Ti-MOFs microflowers was achieved by employing the secondary hydrothermal method, direct hydrothermal reaction of Ti(i-OPr)_4_ (178 mg) and H_4_DOBDC (124 mg) in 10 mL acetic acid was carried out in a 48-h experiment in a 30 mL Teflon-lined stainless-steel autoclave. The produced samples were centrifuged at 8000 rpm for 15 min and rinsed with deionized water and ethanol three times. 

### 2.2. Fluorescence Determination of Aluminium Ion

In a typical procedure, the dispersed solutions were made by combining 200 mL of deionized water with 83.5 mg of Ti-MOFs microflowers, followed by 30 min of ultrasonic agitation. Various concentrations of aluminum ion solutions were prepared in deionized water solution. The fluorescence spectrophotometer excitation wavelength was set at 355 nm, and the excitation and emission slit widths were both 2.0. A volume of 3 mL of the sample in a quartz cuvette was used for the fluorescence measurements. For fluorescence detection of Al(III), 60 μL of Al(III) solutions at various concentrations were individually added to 2940 μL stock solutions of Ti-MOFs microflowers, the final Al(III) concentration range being from 0.4 to 100 μM. 

To investigate the selectivity of the Ti-MOFs microflowers fluorescent probe towards Al(III) determination, various interference ions, such as Cu(II), Cd(II), Ag(I), Co(II), Ni(II), Ca(II), Mg(II), Fe(II), Fe(III), Hg(II), Mn(II), Zn(II), NH_4_^+^, were separately added to the 2970 μL stock solutions of Ti-MOFs microflowers containing 30 μL of 1 mM Al(III). The concentration of all interference ions was 100 μM, i.e., 10-fold higher than that of Al(III), and the same detection conditions were employed as in the aforementioned Al(III) sensing tests.

The following figures and tables provide averages and standard deviations from all selectivity and selectivity studies, which were carried out in triplicate.

### 2.3. Adsorption of Aluminum Ion

For the kinetic adsorption experiment, 300 mg of hierarchical Ti-MOFs microflowers were mixed with 300 mL of 50 mg L^−1^ Al(III) solution. The mixture was agitated at 150 rpm at 25 °C, and the pH of the solution was kept constant at 4.7 ± 0.1 by adding tiny amounts of either HCl (0.1 M) or NaOH (0.1 M). At predetermined intervals, 3 mL samples of the suspension were taken. A 0.22 µm membrane filter was used to filter the samples before inductively coupled plasma optical emission spectrometry (ICP-OES) analysis (ICP-6000, Thermal Electron, Waltham, MA, USA). Thus, the ICP-OES could independently acquire the amount of aluminum present in the Al(III) containing solution. 

Studies of isothermal adsorption were carried out at pH 4.7 ± 0.1 and 25 °C. To attain adsorption equilibration, 20 mg of the as-prepared hierarchical Ti-MOF microflowers were introduced to 20 mL of Al(III) solution (2–100 mgL^−1^) and shaken for 96 h. The aforementioned solution was filtered using a 0.22 µm membrane filter after the liquid and solid phases were separated by centrifugation. The concentration of Al(III) was analyzed by ICP-OES after filtration. The Al(III) adsorption amount was calculated by Equation (1):

(1) $$q_{e}=\frac{\left(C_{0}-C_{e}\right)\times V}{m}$$

where *q_e_* is the amount of Al(III) adsorbed on adsorbent at equilibrium time (mg·g^−1^), and *C*_0_ and *C_e_* represent the initial and equilibrium Al(III) concentrations (mg·L^−1^) in the solution, respectively, *V* indicates the solution volume (mL) and *m* is the mass of the adsorbent (mg).

A pseudo-second-order kinetics model was used to analyze the adsorption kinetic data on the assumption that chemisorption was the rate-determining phase. The pseudo-second-order kinetics mode is presented in Equation (2):

(2) $$\frac{t}{Q}=\frac{1}{Q_{e}}t+\frac{1}{k_{2}Q_{e}^{2}}$$

where *k*_2_ is the pseudo-second-order rate constant (g·mg^−1^h^−1^), *Q_e_* is the equilibrium adsorption capacity (mg·g^−1^), and *Q* is the amount of Al(III) ions adsorbed on the surface of Ti-MOF at time *t* (min), respectively. A linear plot of *t/Q* yields the values of *Q_e_* and *k*_2_.

The capacities of absorbents were determined by equilibrium adsorption isotherms. The experimental data are fitted to Langmuir models.

(3) $$Q_{e}=\;\frac{Q_{m}K_{L}C_{e}}{1+K_{L}C_{e}}$$

where *C_e_* is the equilibrium concentration of Al(III) in aqueous solution (mg·L^−1^), *Q_e_* represents the amount of Al(III) adsorbed on the surface of Ti-MOF (mg·g^−1^), *Q_m_* indicates the maximum amount of Al(III) adsorbed per unit weight of Ti-MOF to form a complete monolayer coverage on the surface, and *K_L_* is the ratio of the rate constants of adsorption and desorption.

## 3. Results and Discussion

**Figure 1:**
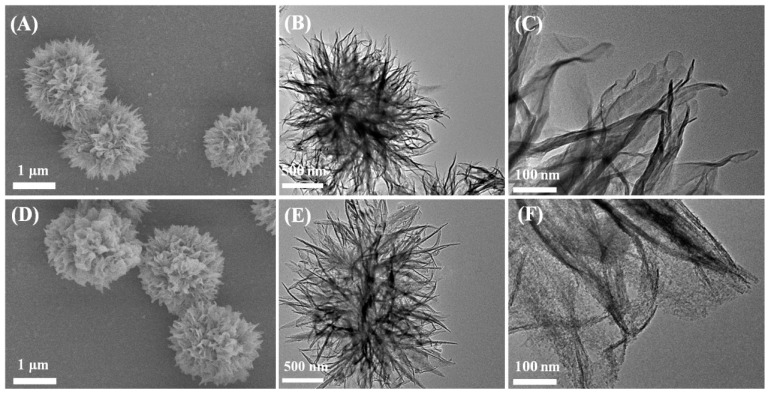
SEM images of Ti(i-OPr)_4−x_/(AcOH)_x_ complex (**A**), and hierarchical Ti-MOF microflowers (**D**). TEM images of Ti(i-OPr)_4−x_/(AcOH)_x_ complex (**B**,**C**), and hierarchical Ti-MOF microflowers (**E**,**F**).

Monodisperse hierarchical Ti-MOF microflowers were produced by a quick, easy, and surfactant-free secondary hydrothermal technique. Through initial hydrothermal synthesis, the precursor of Ti-MOF was fabricated using Ti(i-OPr)_4_ as a metal precursor, and acetic acid as the linker and solvent. Acetate groups (OAc^−^) interact with titanium alkoxides as complexing nucleophilic ligands. Acetate groups function as bidentate bridging ligands during the stoichiometric process, which also results in an increase in the coordination number of Ti from four to six, and the formation of oligomeric species Ti(i-OPr)_4−x_/(AcOH)_x_. Using SEM and TEM measurements, the shape and structure of the Ti(i-OPr)_4−x_/(AcOH)_x_ complex were identified. As illustrated in Figure 1A, the synthesized Ti(i-OPr)_4−x_/(AcOH)_x_ complex exhibited a great number of nanoscale petals combine to produce a monodispersed flower-like hierarchical structure, which typically had a diameter between 1–3 µm. The hierarchical microflowers are shown in a high-magnification TEM image in Figure 1B. It is evident that these microflowers are made up of different 2D nanosheets (petals), which grew from the flower center in all directions to construct 3D hierarchical nanostructures. The petals of the flower-like Ti(i-OPr)_4−x_/(AcOH)_x_ complex have a slightly curved compact structure and an average length of about 500 nm (Figure 1C). It is important to note that the production of the aforementioned Ti(i-OPr)_4−x_/(AcOH)_x_ complex with flower-like hierarchical structures depends critically on acetic acid. By contrast, by using pure deionized water to replace acetic acid, only highly aggregated, irregular particles were produced. Subsequently, the prepared flower-like Ti(i-OPr)_4−x_/(AcOH)_x_ complex was a precursor in a reaction with H_2_DOBDC to produce Ti-MOF through a second hydrothermal synthesis in which AcOH was completely substituted by H_2_DOBDC. The Ti-MOF completely inherited the original flower-like morphology from the Ti(i-OPr)_4−x_/(AcOH)_x_ complex. Interestingly, the petals of Ti(i-OPr)_4−x_/(AcOH)_x_ complex had smooth surfaces, while the petal building block of Ti-MOF, owing to the substitution of acetic acid by H_2_DOBDC, had a perforated structure made of linked or stacked nanoparticles (10–20 nm in size) with a rough surface, as shown in Figure S1. Overall, the microflower morphologies were conserved during the hydrothermal process even if expanding pore channels affected the internal architecture. However, the direct hydrothermal reaction of Ti(i-OPr)_4_ and H_4_DOBDC in acetic acid resulted in pseudo-spherical Ti-MOF nanoparticles 200 ± 20 nm in size (Figure S2). The Ti-MOF microflowers precursor was Ti_2_(HDOBDC)_2_(H_2_DOBDC) (NTU-9) [24]. This has two-dimensional (2D) hybrid layers that are aligned perpendicular to the c-axis in hexagonal prism crystals. Each layer has two-dimensional honeycomb-like layers and is built from the bond between Ti^4+^ and DOBDC ligands. The Ti^4+^ is octahedrally coordinated with six oxygen atoms from the hydroxide and carboxylate groups of the ligand DOBDC, with Ti-O bond lengths of 1.858 and 2.037 Å, respectively. Inside the layers, there are 11 × 11 Å^2^ one-dimensional hexagonal channels that all the uncoordinated oxygen atoms point to. Due to the full exposure of active sites and the low mass transfer diffusion barrier, such a hierarchical, porous structure would, in theory, would be very advantageous for sensing and adsorption processes [19]. 

**Figure 2:**
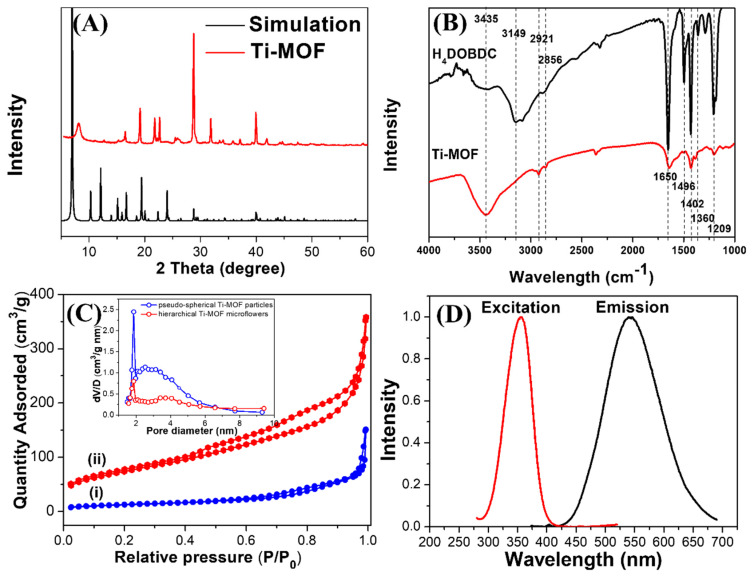
(**A**) Simulated and experimental powder XRD patterns for Ti-MOF microflowers. (**B**) FT-IR spectra of H_4_DOBDC and Ti-MOF microflowers. (**C**) N_2_ adsorption-desorption isotherms and the pore size distribution (inset image) of (i) pseudo-spherical Ti-MOF particles and (ii) hierarchical Ti-MOF microflowers. (**D**) Fluorescence excitation (red line) and emission (blue line) spectra of Ti-MOF microflowers.

The crystalline structure and surface analysis of hierarchical Ti-MOF microflowers were investigated by XRD and FT-IR. As illustrated in Figure 2A, the principal diffraction peaks in the experimental XRD pattern of the hierarchical Ti-MOF microflowers demonstrate a clearly defined crystal structure, and are in good agreement with the simulated patterns of the NTU-9 family [24]. The sharp peaks show the successful fabrication of Ti-MOF and indicate the exceptional crystallinity of the framework. Moreover, the FT-IR measurement was employed to confirm the surface functional groups of Ti-MOF. As illustrated in Figure 2B, Ti-MOF microflowers clearly displayed typical peaks at 3149–3435, 1650, 1496, 1429, 1360 and 1209 cm^−1^ due to the vibrational stretching modes of hydroxyl groups, the C=O group, carbon atoms in the phenyl ring, the O−H group, in-plane O–H bending of phenolic hydroxy, and asymmetric stretching of the C−C−O group, in the same peak position as the H_4_DOBDC ligand. The FT-IR analysis indicates that hierarchical Ti-MOF microflowers are inherently hydrophilic since the structures contain a large number of oxygen-rich functional groups. 

A hierarchical structure with a porous structure is required for an adsorbent to function, and would be beneficial to mass transport. Thus, to characterize the BET surface area and internal pore superstructure of pseudo-spherical Ti-MOF NPs and hierarchical Ti-MOF microflowers, nitrogen adsorption-desorption measurements were performed. As shown in Figure 2C, the isotherm was classified as type IV, with a distinct hysteresis loop at relative pressures ranging from 0.5 to 1.0. The BET-specific surface area of hierarchical Ti-MOF microflowers was estimated to be approximately 264.47 m^2^·g^−1^, which is over 5-fold larger than that of pseudo-spherical Ti-MOF NPs (46.63 m^2^·g^−1^). Moreover, the hysteresis isotherms indicated that Ti-MOF microflowers have a micro/nano-porous structure, which is in line with SEM observations. The hysteresis loop in the Ti-MOF microflowers can be classified as Type H1, indicating a narrow pore size distribution (pore diameter on average: 1.8 nm) according to the Barrett-Joyner-Halenda (BJH) model, as illustrated in the inset of Figure 2C. 

To further investigate the optical properties of Ti-MOF microflowers, fluorescence spectrum studies were carried out. As shown in Figure 2D, the maximal excitation and emission peaks were located at 355 and 538 nm, respectively. A large Stokes shift of 187 nm occurred in the Ti-MOF microflowers to avoid crosstalk between excitation and emission signals. The quantum yield of Ti-MOF microflowers was estimated at 7.49%. Furthermore, due to their insufficient water solubility, most Al(III)-selective fluorescence chemosensors are studied in pure organic (such as DMSO and THF) or organic-water mixed solutions [25]. Therefore, the effects of various conditions on Ti-MOF microflowers should be investigated in order to determine fluorescence stability. As displayed in Figure S3, no distinct photobleaching under UV lamp irradiation over 5 h was observed, demonstrating the remarkable optical stability of Ti-MOF microflowers compared to traditional fluorescent dyes. The fluorescence properties of Ti-MOF microflowers were also investigated at different ionic strengths (modulated by 0–1000 mM NaNO_3_) and different pH (4.0–9.0) conditions, as illustrated in Figure S4. There was no discernible change in fluorescence intensity in aqueous solutions under different ionic strengths and pH conditions, indicating that Ti-MOF microflowers are extremely stable even under high ionic strength and extreme pH conditions. Moreover, the Ti-MOF microflowers solution remained homogenous for over 1 month at room temperature (without any aggregation or color change). Their excellent water solubility and optical stability makes them potential candidates for a new class of fluorophores for determination of metal ions in aqueous solution without organic co-solvents.

**Figure 3:**
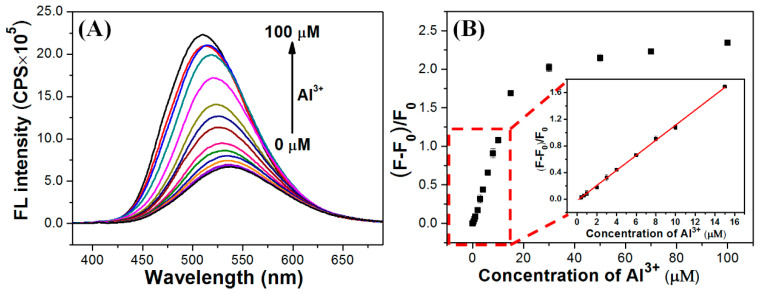
(**A**) Fluorescence response of Ti-MOF microflowers with various concentration of Al(III) (from bottom to top: 0, 0.4, 0.7, 1, 2, 3, 4, 6, 8, 10, 15, 30, 50, 70, 100 μM). (**B**) Fluorescence enhancement rate ((F−F_0_)/F_0_ at 510 nm) of Ti-MOF microflowers to Al(III) at various concentrations (0 to 100 μM). The linear detection range for 0.4–15 μM. Al(III) is shown in the inset image. The error bars represent standard deviations from three independent measurements.

Owing to their excellent fluorescence property and water stability, Ti-MOF microflowers are proposed to be used as fluorescent probes for quantitative determination of Al(III) ions. Because response rate is important in sensing performance, the time-response characteristics of hierarchical Ti-MOF microflowers towards Al(III) were investigated, in which the fluorescence signals of the reaction mixture were collected immediately and consecutively upon the addition of 10 M Al(III) into the Ti-MOF microflowers suspension. Figure S5 shows the difference in FL intensity before and after addition of 10 M Al(III). The fluorescence intensity of Ti-MOF microflowers significantly increased within 2 min after Al(III) addition and remained essentially constant afterward, indicating that the Al(III)-induced fluorescence enhancement reaction is quick. The FL intensity-Al(III) concentration ([Al(III)]) relationship was then established. Figure 3 shows the change of fluorescence intensity of Ti-MOF microflowers with gradually increasing Al(III) concentration from 0 to 100 μM for evaluating their fluorescence sensitivity. Figure 3A shows that the maximum emission peak of Ti-MOF microflowers appears at 538 nm, and is blue-shifted to 510 nm with remarkable fluorescence enhancement with increasing concentrations of Al(III). Figure 3B shows a plot of fluorescence enhancement rate (FER, derived from the peak intensity data in Figure 3A) against Al(III) concentration. There is an excellent linear correlation (Y = 0.114X − 0.024, R^2^ = 0.999) between the FER and [Al(III)] in the low concentration range of 0.4–15 μM. The real limit of detection (LOD) is 0.4 µM, based on Figure 3B. Furthermore, the maximum FER could reach 2.34 as the concentration of Al(III) increased to 100 μM. On the basis of a signal-to-noise ratio of 3σ/κ (κ: slope; σ: standard error), the theoretical LOD was estimated to be 75 nM, which is approximately 100-fold lower than the permitted level (7.4 μM) of aluminum in drinking water by the WHO. Ti-MOF analytical performance is comparable to, or better than, that of previously reported FL sensors for Al(III) detection, as shown in Table S1. The poor coordination ability and strong hydration ability of Al(III) has hampered the development of a suitable fluorescence sensor [26]. A Job’s plots experiment was carried out to demonstrate the coordination of Al(III) and Ti-MOF microflowers. The maximum fluorescence intensity as a function of molar fraction of Al(III) was used in the Job’s method, and the total concentration of Ti-MOF and Al(III) ion was 20 μM. As shown in Figure S6, 3:2 stoichiometric complexations between Ti-MOF microflowers and Al(III) was confirmed. 

**Figure 4:**
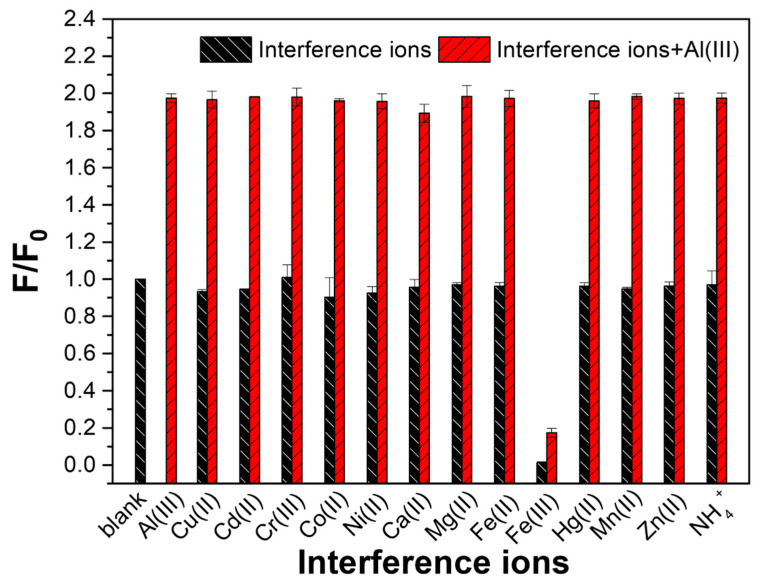
Ion selectivity ability of Ti-MOF microflowers towards various cations. The blank and Al(III) bar represent the Ti-MOF microflowers and the Ti-MOF microflowers with 10 μM Al(III), respectively. The black bars represent the Ti-MOF microflowers with a variety of single interference ions (100 μM). The red bars indicate the Ti-MOF microflowers with mixtures containing 10 μM Al(III) and 100 μM various interference ions. F_0_ and F denote FL intensity of the blank in the absence and presence of the target ions, respectively.

Because of the similar electron configuration in the recognition process, other trivalent ions such as Fe^3+^ and Cr^3+^ frequently interfere with Al(III) detection [27]. Thus, Al(III)-selective fluorescent probes are still in high demand. The Al(III) ion selective detection ability of Ti-MOF microflowers was evaluated towards different interference ions including Cu(II), Cd(II), Cr(III), Co(II), Ni(II), Ca(II), Mg(II), Fe(II), Fe(III), Hg(II), Mn(II), Zn(II) and NH_4_^+^ under similar conditions. The fluorescence intensity ratios (F/F_0_) of Ti-MOF microflowers in the absence and presence of interference metal ions are shown in Figure 4. As expected, the addition of 10 μM Al(III) resulted in over a two-fold FL enhancement compared with that of the blank. Except for Fe^3+^ ions, there was no significant change in FL intensity based on emission at 510 nm for all interference ions at 10-fold higher concentrations than Al(III). Subsequently, the fluctuation of the F/F_0_ was recorded after 10 μM of Al(III) was injected into the above solution to form a competing mixture. The obtained value of the F/F_0_ after the solution was added with Al(III), as shown by the green bars in Figure 4, was consistent with the control group, in which Ti-MOF microflowers were simply mixed with Al(III). Furthermore, Fe(III) ions could reduce the fluorescence of Ti-MOF microflowers, while Fe(II) ions resulted in no change in FL intensity of Ti-MOF microflowers. Thus, we employed reductant (e.g., ascorbic acid, AA) to reduce Fe(III) ions to shield the interference of Fe(III) ions towards FL detection of Al(III) ions by Ti-MOF microflowers. As illustrated in Figure S7A, the fluorescence response of Ti-MOF microflowers to Al(III) ions was unaffected by AA. Due to the reduction of Fe(III) ions by AA, the fluoresce intensity of Ti-MOF with Fe(III) ions in the presence of AA showed no significant difference compared to the control group (Figure S7B). All in all, the Al(III) induced fluorescence enhancement of Ti-MOF was not obviously influenced by the interference ions, and further illustrates that Ti-MOF microflowers display an extremely selective response to Al(III) ions.

### 3.1. Removal of Al(III) by Ti-MOF Microflowers

**Figure 5:**
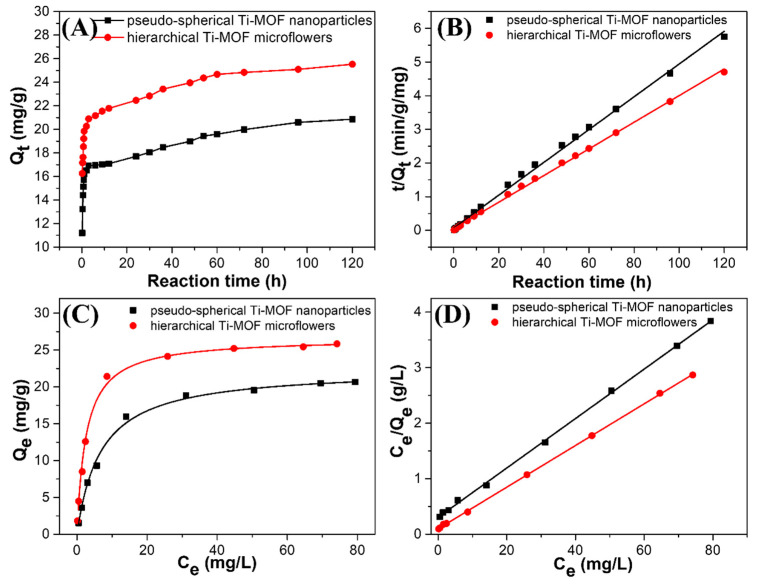
(**A**) Kinetics of Al(III) adsorption and (**B**) the corresponding fitting curves for pseudo-spherical Ti-MOF particles and hierarchical Ti-MOF microflowers via a pseudo-second-order kinetic model (50 mg·L^−1^ of the initial concentration of Al(III), 1 g·L^−1^ of adsorbents with initial solution pH 4.7 ± 0.1 at 25 °C). (**C**) Isothermal adsorption of Al(III), and (**D**) the corresponding plots of *C_e_/Q_e_* versus *C_e_* for pseudo-spherical Ti-MOF nanoparticles and hierarchical Ti-MOF microflowers (2–100 mg·L^−1^ of initial Al(III) concentration, 1 g·L^−1^ of adsorbents, 96 h of equilibrium time at 25 °C and initial pH 4.7 ± 0.1).

In addition to accurate and dependable detection, effective removal of Al(III) from polluted water is critical in wastewater treatment to reduce Al(III) accumulation below levels that pose a serious threat to humans. Time-dependent Al(III) adsorption kinetics experiments were performed to gain insight into the adsorption behavior of Ti-MOF microflowers towards Al(III), with an initial concentration of Al(III) set at 50 mgL^−1^ and a sorbent dosage of 1 g·L^−1^. To reveal the outstanding characters of the hierarchical structure, the adsorption performance of Ti-MOF microflowers was contrasted with that of pseudo-spherical Ti-MOF nanoparticles synthesized by a direct hydrothermal reaction. As illustrated in Figure 5, it is clear that the concentration of Al(III) decreased rapidly at the beginning (2 h), and then approached a constant value after a long contact time (approximately 60 h) for both types of Ti-MOF. In the case of hierarchical Ti-MOF microflowers, the adsorption of Al(III) was as high as 21.78 mg·g^−1^ within just 12 h. With increasing treatment time, the adsorption of Al(III) on the hierarchical Ti-MOF microflowers was increased to 25.5 mg·g^−1^, which is higher than that of pseudo-spherical Ti-MOF nanoparticles (20.8 mg·g^−1^). A pseudo-second-order kinetic model was used to describe the kinetic characteristics of the adsorption sites, which was proportional to the square of the number of unoccupied sites, according to Equation (2), and as shown in Figure 5B. The parameters *Q_e_* and *k_2_* for Al(III) adsorption were estimated by using linear fitting with high correlation coefficients (R^2^ = 0.998), as shown in Table 1. The low value of *k*_2_ indicates that the removal rates of Al(III) by both type of Ti-MOF were fast. Furthermore, *h=k*_2_*Q_e_*^2^ was calculated to quantitatively describe the initial removal rate using Equation (1). The initial removal rate (*h*) of Al(III) on the hierarchical Ti-MOF microflowers was larger than that of pseudo-spherical Ti-MOF nanoparticles, demonstrating a higher removal rate of Al(III) by hierarchical Ti-MOF microflowers than that of pseudo-spherical Ti-MOF nanoparticles. These results might be attributed to the distinctive hierarchical structure of Ti-MOF microflowers, the thin nanosheets reducing the diffusion resistance of Al(III) ions and allowing for faster kinetics.

Isothermal adsorption experiments were carried out based on kinetic adsorption behavior, and a reaction time of 96 h was used for the equilibrium adsorption time. Figure 5C shows the adsorption isotherm of Al(III) on pseudo-spherical Ti-MOF nanoparticles and hierarchical Ti-MOF microflowers by varying initial Al(III) concentrations between 2–100 mg·L^−1^ under initial solution pH of 4.7 ± 0.1. Adsorption of Al(III) increased with increasing concentrations of Al(III) in aqueous solution for both types of Ti-MOF. Table 1 shows the fitted results and calculated parameters for the Langmuir model, which was used to further analyze the Al(III) adsorption isotherms. Obviously, the Al(III) adsorption results for the two adsorbents with a high correlation coefficient, could be well fitted by the Langmuir model (R^2^ = 0.999), which applies to monolayer adsorption on a surface with a finite number of identical active sites. Figure 5D shows the corresponding plots, which indicate good linear relationships for both types of Ti-MOF. The substantial maximum Al(III) removal capacities (q_m_) were up to 20.66 mg·g^−1^ for pseudo-spherical Ti-MOF nanoparticles and 25.85 mg·g^−1^ for hierarchical Ti-MOF microflowers. Based on the Langmuir model these outperformed the adsorption capacities of other adsorbents listed in Table S2. The superior adsorption capacity of hierarchical Ti-MOF microflowers towards Al(III) can be attributed to their higher specific surface area in comparison to pseudo-spherical Ti-MOF nanoparticles, as well as their unique hierarchically flower-like morphology composed of ultrathin nanosheets that provide abundant adsorption sites for Al(III) capture. 

### 3.2. Possible Mechanism for Selective Determination of Al(III) Ions

According to previous research [28], the effect of metal cations on luminescent MOFs can be attributed to three factors: (1) the collapse of the MOFs framework, (2) cation exchange between the MOFs’ central cations and the target cations, and (3) interactions between the metal cations and organic ligands. XRD and SEM measurements were used to confirm whether the crystal structure of the original framework was affected in order to further understand and elucidate the possible sensing mechanism of the phenomenon, in which Al(III)-enhances the FL of Ti-MOF microflowers. The XRD patterns (Figure S8) and SEM images (Figure S9) revealed that the sample obtained by immersing Ti-MOF microflowers in Al(III) ions was nearly identical to the original Ti-MOF, indicating that the Al(III) ions do not cause the Ti-MOF framework to collapse or change.

To verify whether the cation exchange between the central cations of Ti-MOFs and Al(III) occurred, ICP-OES was used to determine the concentrations of Ti(IV) and Al(III) in the solid MOF and supernatant after Ti-MOF treatment with various concentrations of Al(III) ions. As illustrated in Table S3, there were no Ti(IV) in all supernatants after Ti-MOF treatment with different concentration of Al(III) ions, which precluded the possibility of cation exchange between Ti(IV) and Al(III). However, Al(III) was found in all solid Ti-MOF after treatment with different concentrations of Al(III) ions. The summation of Al(III) ions in the supernatant and solid Ti-MOF were equal to the initial concentration of Al(III) ions, which is consistent with the results of the adsorption experiment. This verifies that Ti-MOF microflowers can form a complex with Al(III) ions. 

**Figure 6:**
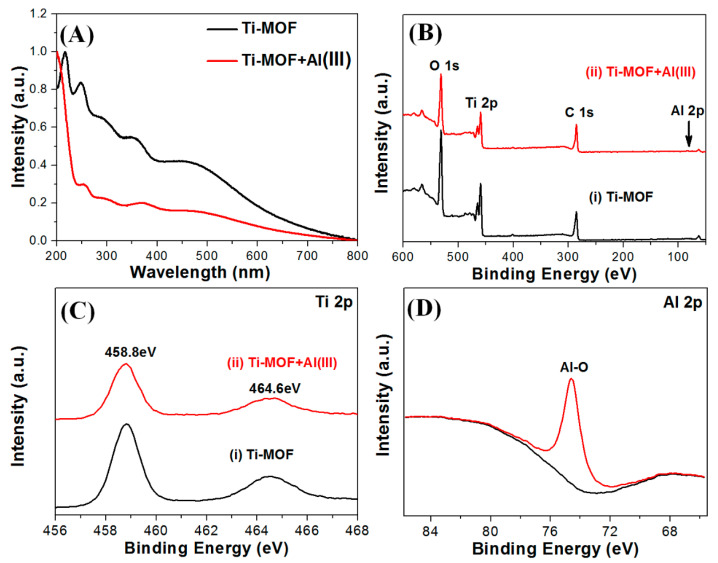
(**A**) UV-vis absorption spectra of Ti-MOF microflowers before and after Al(III) addition. (**B**) XPS spectra of Ti-MOF microflowers before and after Al(III) addition. (**C**) Ti2p spectra of Ti-MOF microflowers before and after adding Al(III). (**D**) Al2p spectra of Ti-MOF microflowers after addition of Al(III).

Because of the interaction between metal cations and organic ligands, we propose that the Al(III)-induced fluorescence enhancement of Ti-MOF microflowers can be attributed to the chelation-enhanced fluorescence (CHEF) mechanism [29]. As previously stated, FT-IR spectral analysis shows that the prepared Ti-MOF microflowers have phenolic hydroxyl and carboxyl groups. Metal ions can be chelated by these functional groups to form stable coordination complexes. The fluorescent selectivity of Ti-MOF microflowers towards Al(III) may be attributed to the smaller ionic radius (0.5 Å) of Al(III), which allows for a suitable coordination geometry with Ti-MOF, and a higher charge density, causing strong coordination between Al(III) and Ti-MOF. After adding Al(III), the carbonyl O, and hydroxyl O on DOBDC of Ti-MOF microflowers can coordinate with the Al(III) center atom, which increases the energies of the n–π* transitions compared to the corresponding π–π* transitions, and the photoinduced electron transfer process is interrupted, and the FL enhanced [30,31]. As illustrated in Figure 3A, the Al(III) ions induced a blue-shift with remarkable fluorescence enhancement of Ti-MOF microflowers. The wavelength shift indicates that the structure of DOBDC ligands in the surface of Ti-MOF microflowers may be changed in the presence of Al(III), which could result from phenolic hydroxy and carboxyl of DOBDC strongly bound with Al(III). Figure 6A shows the UV-vis absorption spectra of Ti-MOF microflowers in the present of Al(III) ions. Ti-MOF microflowers have four absorption peaks at 248, 290, 350, 458 nm, while all of four absorption peaks are red-shifted (253, 291.5, 368.5 and 486 nm) after adding Al(III) ions. It is well known that the binding of an auxochrome to a chromophore causes increased absorption and a red shift of the chromophore. This further illustrates that Ti-MOF microflowers can form a complex with Al(III) ions. 

The coordination interaction between the Al(III) and the carbonyl O, and hydroxyl O on DOBDC of Ti-MOF microflowers was further validated by XPS. Figure 6B–D shows the survey spectra, Ti 2p and Al 2p spectra of Ti-MOF microflowers in the absence and present of Al(III). According to the XPS spectra, the binding energies around 286, 459, 531 eV corresponded to the C 1s, Ti 2p and O 1s, which are the basic elements of the Ti-MOF microflowers (Figure 6B). After the interaction with Al(III), the emergence of the Al 2p peak at 74.6 eV (Al-O) verified that Al(III) is loaded into the framework of Ti-MOF microflowers (Figure 6C). 

## 4. Conclusions

A secondary hydrothermal method was used to successfully prepare three-dimensional hierarchical titanium-based metal-organic framework (Ti-MOF) microflowers for the simultaneous detection and removal of Al(III) ions in aqueous solution. The as-prepared Ti-MOF microflowers are made up of flowerlike micro/nanostructured particles with sizes ranging from 5 to 6 μm. The particles are made of 200 nm nanorods with a length of 1–2 µm and a porous structure with a pore diameter of 1.8 nm and a specific surface area of 264.47 m^2^·g^−1^, which provides abundant surface active sites for detection and adsorption of Al(III) ions. The resultant Ti-MOF microflowers exhibit excellent stability, high water dispensability, and dramatical fluorescence characteristic, making them useful as fluorescence probes for quantitative detection of Al(III) in aqueous solution. Importantly, specific FL enhancement by Al(III) via the chelation-enhanced fluorescence (CHEF) mechanism can be utilized to selectively and accurately determine Al(III) due to the coordination interaction between the Al(III) and the carbonyl O, and hydroxyl O on DOBDC of Ti-MOF microflowers. Linear concentration ranges of up to 15 μM and a detection limit of 75 nM are easily achievable, the latter being approximately 100-fold lower than the WHO permitted level of aluminum in drinking water (7.4 μM). By introducing ascorbic acid, the interference of Fe(III) can be shielded to achieve selective detection of Al(III) in the presence of various cations. Ti-MOF microflowers have an excellent adsorption capacity for Al(III), with a maximum adsorption capacity of 25.85 mg·g^−1^. The adsorption rate is rapid and corresponds to a pseudo-second-order kinetic model. The Ti-MOF is an excellent candidate as a fluorescent chemical sensor and adsorbent for aqueous contaminants due to its impressive water stability, high porosity, and strong luminescence.

## Figures and Tables

**Table 1:** Adsorption parameters of Al(III) on both type of Ti-MOF.

Adsorbents	Kinetic Adsorption (Pseudo-Second-Order Model)		Isotherms (Langmuir Model)		
	K_2_ (g·mg^−1^· min^−1^)	*Q_e_* (mg·g^−1^)	*h* (mg·g^−1^ ·min^−1^)	q_m_ (mg·g^−1^)	*K_L_* (L·mg^−1^)
pseudo-spherical Ti-MOF NPs	0.0324	20.86	13.67	20.66	0.1468
hierarchical Ti-MOF microflowers	0.0343	25.53	21.92	25.85	0.3831

## Data Availability

Not applicable.

## Supplementary Materials

The following supporting information can be downloaded at: https://www.mdpi.com/article/10.3390/bios12110935/s1, Figure S1: HRTEM images of hierarchical Ti-MOF nanoflowers through secondary hydrothermal synthesis; Figure S2: SEM image of pseudo-spherical Ti-MOF by direct hydrothermal reaction; Figure S3: Photostability of Ti-MOF nanoflowers under UV lamp irradiation; Figure S4: Relative fluorescence intensity (F/F_0_) of Ti-MOF nanoflowers at different ionic strengths (0–1000 mM NaNO_3_) and pH values (4.0–9.0); Figure S5: Fluorescence response—time profile of Ti-MOF nanoflowers before and after adding 10 μM Al(III); Figure S6: Job’s plot for stoichiometric determination of Ti-MOF and Al(III); Figure S7: Fluorescence spectra of Ti-MOF in the presence of Al^3+^ and AA, and Fluorescence spectra of Ti-MOF in the presence of Fe^2+^, Fe^3+^ and AA; Figure S8: PXRD pattern of Ti-MOF nanoflowers after treatment with Al^3+^ ions; Figure S9: SEM image of Ti-MOF nanoflowers after treatment with Al^3+^ ions; Table S1: Comparison of different fluorescent material sensors for Al(III) determination [32,33,34,35,36,37,38,39,40,41,42,43,44]; Table S2: Comparison of the Al(III) adsorption capacities for different adsorbent materials [35,45,46,47,48,49,50,51]; Table S3: ICP-OES analysis of the content of Ti^4+^ and Al^3+^ in the solid MOF and supernatant after Ti-MOF treatment with different concentration of Al^3+^ ions.
