# Supplementary material for: Hierarchical Ti-MOF Microflowers for Synchronous Removal and Fluorescent Detection of Aluminum Ions
Source: Biosensors (Basel). 2022 Oct 27;12(11):935. doi: 10.3390/bios12110935 (PMC9688045; doi:10.3390/bios12110935)
Supplement: Supplementary file 1 [file biosensors-12-00935-s001.zip › biosensors-1987785-supplementary.pdf]

Supplementary Information

Hierarchical Ti-MOF nanoflowers for synchronous removal and fluorescent detection of aluminum ions

Jianguo Zhou,[a,b,†] Jieyao Song,[b,c †] Guangqiang Ma,[d] Yongjian Li,[b] Yanan Wei,[b] Fei Liu,[b] Hongjian Zhou[a,b,c*]

[a] Institutes of Physical Science and Information Technology, Anhui University, Hefei, 230601, P. R. China.

[b] Key Laboratory of Materials Physics, Centre for Environmental and Energy Nanomaterials, Anhui Key Laboratory of Nanomaterials and Nanotechnology, Institute of Solid State Physics, HFIPS, Chinese Academy of Sciences, Hefei 230031, P.R. China.

[c] Department of Materials Science and Engineering, University of Science and Technology of China, Hefei 230026, P. R. China.

[d] School of Mechanical and Electrical Engineering, Xi'an University of Architecture and Technology, Xi'an 710055, P. R. China.

[†] These authors contributed equally to this work.

[*]Corresponding author: hjzhou@issp.ac.cn (H. Zhou); Tel: 0086-55165596305; Fax: 0086-55165591434.

Experimental section

Reagents and Materials

$Ti(i\text{-}OPr)_4$ (titanium isopropoxide) and H_4DOBDC (2,5-dihydroxyterephthalic acid) were purchased from Aladdin Reagent Co. Ltd. (Shanghai, China). Acetic acid, ethanol, and $Al(NO_3)_3{\cdot}9H_2O$ were purchased from the Sinopharm Chemical Reagent Co. Ltd. (Shanghai, China). Milli-Q Milipore 18.2 MΩ.cm water was used as the solvent throughout the whole experiments. All other metal ions were prepared from their nitrate, sulfate, or chloride salts.

Characterization

The as-synthesized Ti-MOFs nanoflowers were characterized by scanning electron microscopy (SEM, FEI Sirion 200), High resolution transmission electron microscopy (HTEM, JEOL-2010 at 200 kV). The phase structures of the products were characterized by X-ray diffractometer (Philips, X'Pert-PRO, Netherlands) with using the Ni-filtered monochromatic Cu-Kα radiation (λKα1=1.5418 Å) at 40 keV and 40 mA. X-ray Photoelectron Spectroscopy (XPS) analysis were performed on an Thermo Scientific ESCALAB 250 X-ray photoelectron spectrometer (Thermo, America) with Al $K\alpha_{1,2}$ monochromatized radiation at 1486.6 eV X-rays source. Fourier transformed infrared (FT-IR) spectra were measured on a Thermo Nicolet NEXUS FI-IR spectrophotometer (Thermo-Nicolet Inc., America). Fluorescence spectra for the samples were acquired by a FluoroMax-4 fluorescence spectrophotometer (Horiba Jobin Yvon Inc., France). The luminescence decay profile of the sample was obtained by a time-correlated single-photon-counting (TCSPC) technique under a lifetime fluorescence spectrometer (DeltaFlex, Horiba Jobin Yvon, France) equipped with a 370 nm NanoLED as an excitation source (200 ps pulse duration, HORIBA). The UV-vis absorption spectra were carried out with a UV-2700 spectrophotometer (Shimadzu, Japan). All pH measurements were made with a S220 SevenCompact™ pH meter (Mettler-Toledo Instruments (Shanghai) Co. Ltd., China).

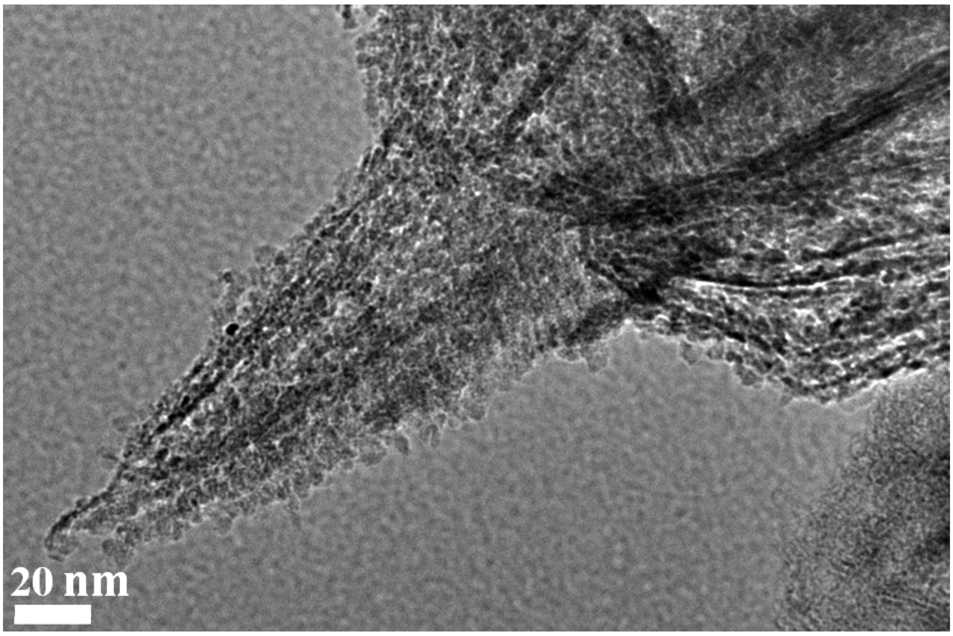

Figure S1. HRTEM images of hierarchical Ti-MOF nanoflowers through secondary hydrothermal synthesis

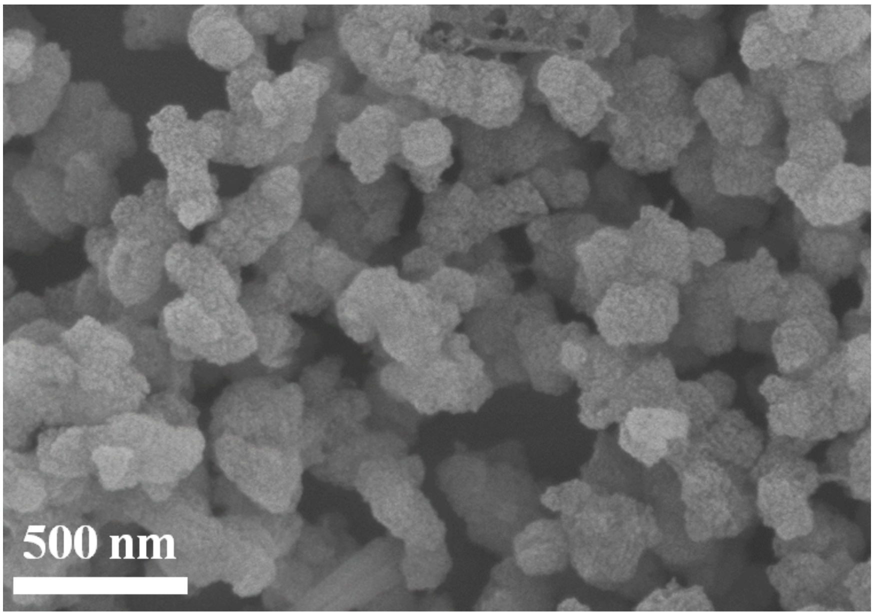

Figure S2. SEM image of pseudo-spherical Ti-MOF by direct hydrothermal reaction

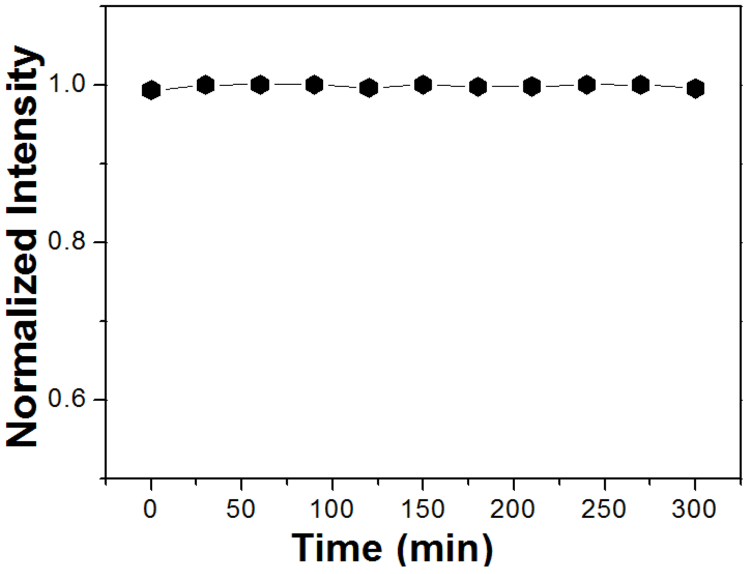

Figure S3. Photostability of Ti-MOF nanoflowers under UV lamp irradiation.

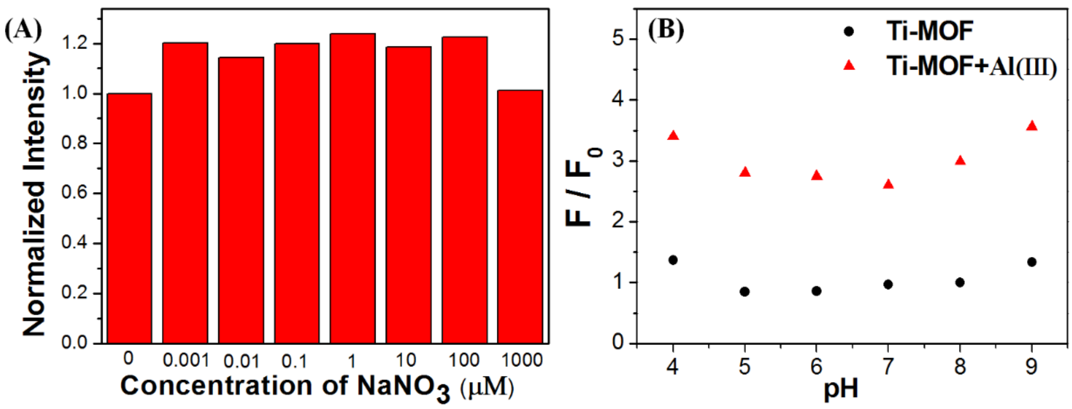

Figure S4. Relative fluorescence intensity (F/F_0) of Ti-MOF nanoflowers at (A) different ionic strengths (0–1000 mM $NaNO_3$) and (B) pH values (4.0–9.0)

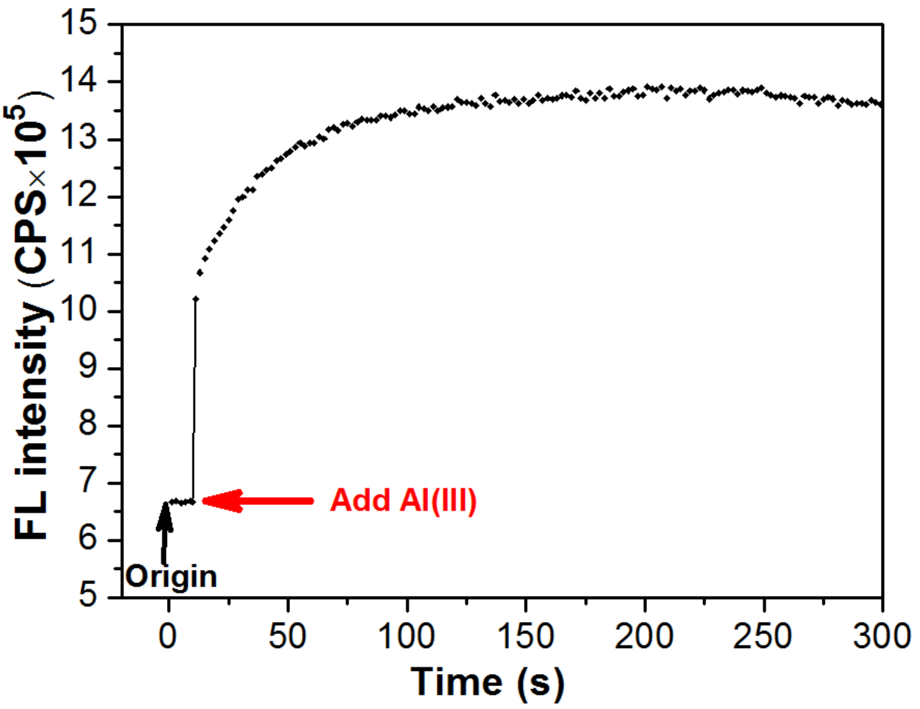

Figure S5. Fluorescence response – time profile of Ti-MOF nanoflowers before and after adding 10 μM Al(III).

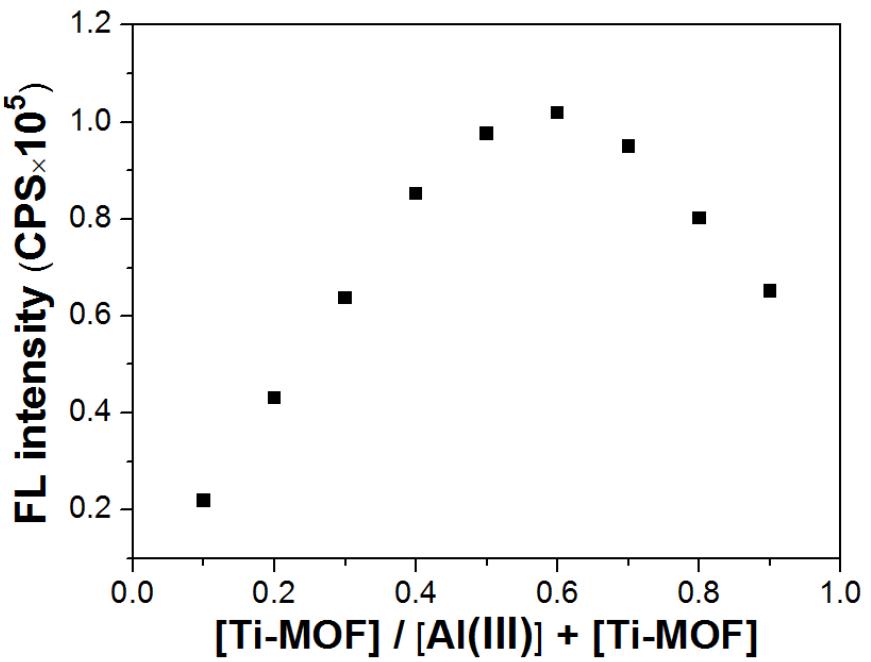

Figure S6. Job's plot for stoichiometric determination of Ti-MOF and Al(III)

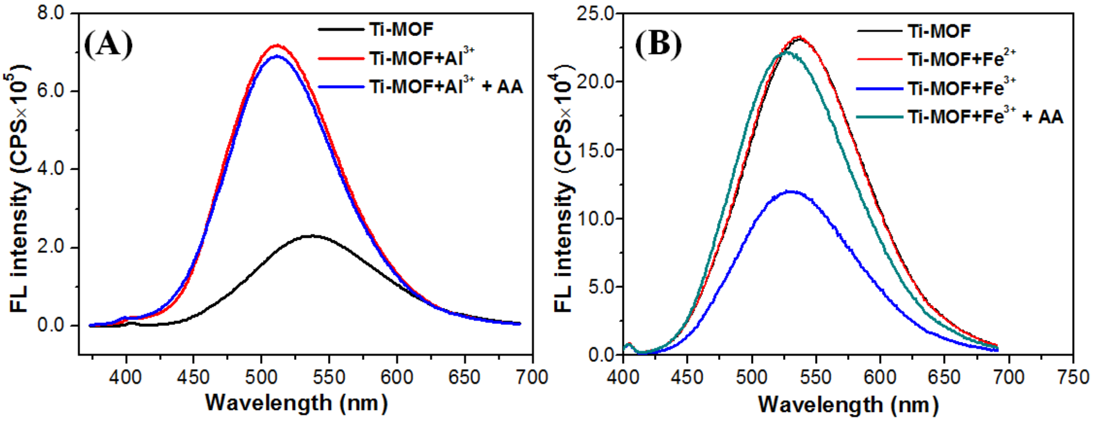

Figure S7. (A) Fluorescence spectra of Ti-MOF in the presence of Al^{3+} and AA, and (B) Fluorescence spectra of Ti-MOF in the presence of Fe^{2+}, Fe^{3+} and AA.

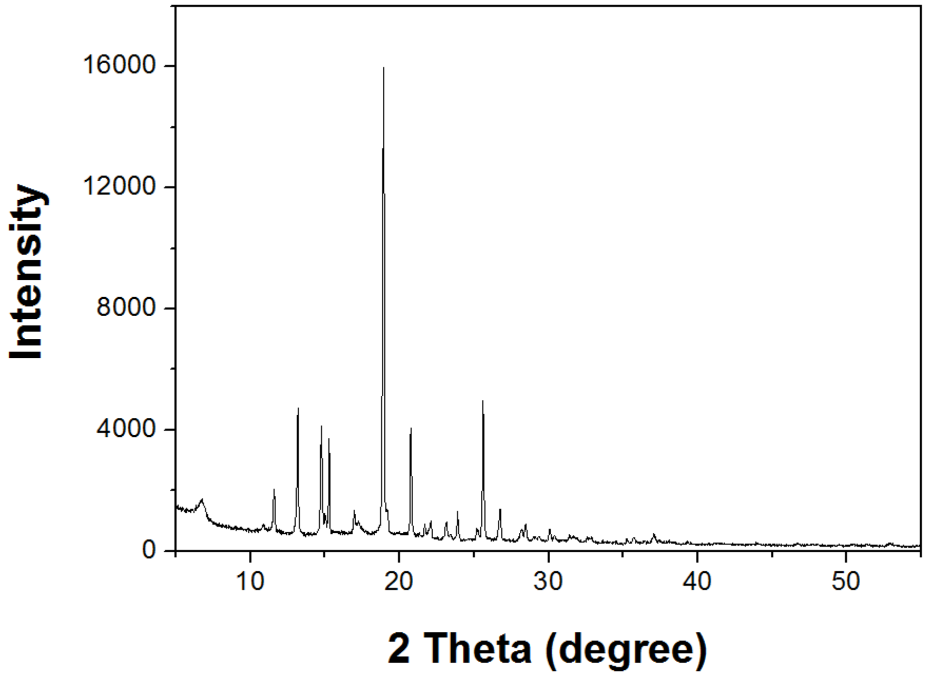

Figure S8. PXRD pattern of Ti-MOF nanoflowers after treatment with Al^{3+} ions

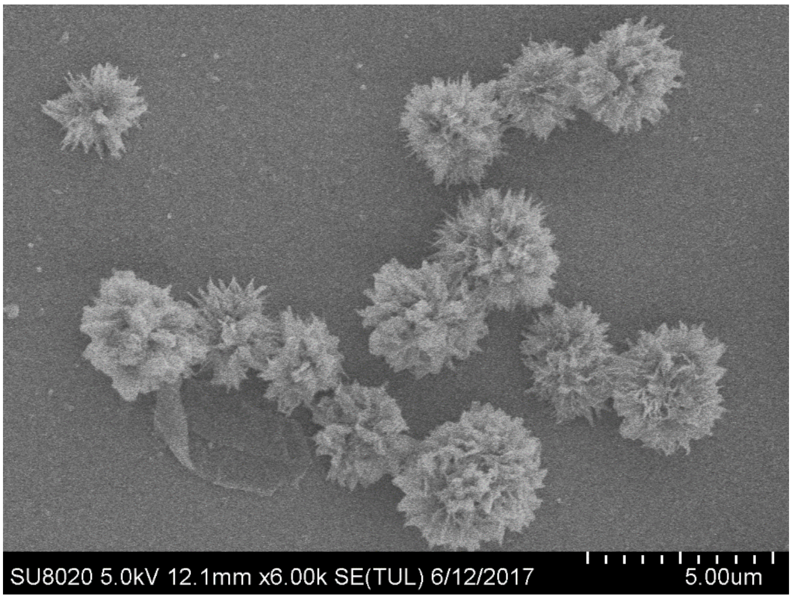

Figure S9. SEM image of Ti-MOF nanoflowers after treatment with Al^{3+} ions

Table S1. Comparison of different fluorescent material sensors for Al(III) determination.

Fluorescence sensors	Linear range (μM)	Detection limit (μM)	Responsive time	Reference
MSA-AgAu NCs	2 – 30	0.8	5 min	[1]
RG-HN	0.2 – 1.4	0.026	–	[2]
PFE/sodium citrate complex	0.5 – 9	0.37	–	[3]
RBMSiO$_2$-20	20 − 100	0.13	–	[4]
Coumarin-based fluorescent probe	0 – 10	0.1	–	[5]
3D Cd-MOF	0 – 50	0.56	30 seconds	[6]
((*E*)-*N′*-((8-hydroxy-1,2,3,5,6,7-hexahydropyrido[3,2,1-*ij*]quinolin-9-yl)methylene)benzohydrazide)	50 – 110	0.193	–	[7]
Paper-based strips of c-dots-R6G	0 – 1000	38.9	5 min	[8]
Salicylimine (L)	0 – 10	0.0294	–	[9]
$[Co_2(dmipm)(nda)_2]_n$	35 – 150	0.7	seconds	[10]
Rhodamine-Furan type chemosensor (RF)	2 – 24	1.6	15 min	[11]
DTT-CuNCs	0.01 – 7	0.01	1 min	[12]
Tyrosine-Au NCs	0 – 1000	0.3	5 min	[13]

**hierarchical Ti-MOF nanoflowers**	**0 – 15**	**0.075**	**2 min**	**This work**

Table S2. Comparison of the Al(III) adsorption capacities for different adsorbent materials

Adsorbents	Adsorption capacity (mg g^{-1})	Experimental condition		Reference
		pH	*T*(K)	
Streptomyces rimosus biomass	11.76	4–4.25	298	[14]
$RBMSiO_2$-20	23.2	–	–	[4]
Fe_3O_4/TEOS/AMEO/GA	24.8	5.0	308	[15]
Beach cast seaweed	22.5	9±0.1	298	[16]
BDH activated carbon	6.562	4.0	295	[17]
MIP beads	3.315	5.0	293	[18]
GBH functionalized XAD-16 resin	24.28	9±0.01	300±0.2	[19]
PAN-based adsorbents	0.71	5.6	293	[20]
**pseudo-spherical Ti-MOF NPs**	**20.86**	**4.7**	**298**	**This work**
**hierarchical Ti-MOF nanoflowers**	**26.65**	**4.7**	**298**	**This work**

Table S3. ICP-OES analysis of the content of Ti^{4+} and Al^{3+} in the solid MOF and supernatant after Ti-MOF treatment with different concentration of Al^{3+} ions.

Initial concentration of Al^{3+}	Samples	Al^{3+} (ppm)	Ti^{4+}(ppm)
10 ppm	supernatant	2.24	0.14
	solid MOF	7.91	99.77
20 ppm	supernatant	9.05	0.18
	solid MOF	11.07	100.23
50 ppm	supernatant	25.5	0.25
	solid MOF	24.9	101.08

References

1. Zhou, T.-y., et al., *Silver–gold alloy nanoclusters as a fluorescence-enhanced probe for aluminum ion sensing.* Analytical chemistry, 2013. **85**(20): p. 9839-9844.
2. Ku, K.-S., et al., *A new rhodamine 6G based chemosensor for trace level Al^{3+} and its thin film application in 100% aqueous medium.* Sensors and Actuators

B: Chemical, 2016. **236**: p. 184-191.
3. Wang, H., et al., *Citrate-induced aggregation of conjugated polyelectrolytes for Al3+-ion-sensing assays.* ACS applied materials & interfaces, 2013. **5**(16): p. 8254-8259.
4. Xu, L., et al., *Rhodamine B-based ordered mesoporous organosilicas for the selective detection and adsorption of Al (III).* New Journal of Chemistry, 2016. **40**(8): p. 6752-6761.
5. Xiao, H., et al., *A highly selective turn-on fluorescent probe for Al (III) based on coumarin and its application in vivo.* Analyst, 2014. **139**(8): p. 1980-1986.
6. Lv, R., et al., *A highly selective and fast-response fluorescent probe based on Cd-MOF for the visual detection of Al^{3+} ion and quantitative detection of Fe3+ ion.* Journal of Solid State Chemistry, 2018. **259**: p. 67-72.
7. Lee, S.A., et al., *A new multifunctional Schiff base as a fluorescence sensor for Al3+ and a colorimetric sensor for CN- in aqueous media: an application to bioimaging.* Dalton Transactions, 2014. **43**(18): p. 6650-6659.
8. Kim, Y., G. Jang, and T.S. Lee, *New fluorescent metal-ion detection using a paper-based sensor strip containing tethered rhodamine carbon nanodots.* ACS applied materials & interfaces, 2015. **7**(28): p. 15649-15657.
9. Liang, C., et al., *A highly selective fluorescent sensor for Al^{3+} and the use of the resulting complex as a secondary sensor for PPi in aqueous media: its applicability in live cell imaging.* Dalton Transactions, 2015. **44**(25): p. 11352-11359.
10. Chen, W.-M., et al., *A superior fluorescent sensor for Al^{3+} and UO_2^{2+} based on a Co (ii) metal–organic framework with exposed pyrimidyl Lewis base sites.* Journal of Materials Chemistry A, 2017. **5**(25): p. 13079-13085.
11. Mi, Y., et al., *A reversible switch as highly selective sequential chemosensor for Al^{3+} cation followed by F^{-} anion.* Sensors and Actuators B: Chemical, 2014. **192**: p. 164-172.
12. Hu, X., et al., *One-step synthesis of orange fluorescent copper nanoclusters for sensitive and selective sensing of Al^{3+} ions in food samples.* Sensors and Actuators B: Chemical, 2017. **247**: p. 312-318.
13. Mu, X., et al., *One-pot synthesis of tyrosine-stabilized fluorescent gold nanoclusters and their application as turn-on sensors for Al^{3+} ions and turn-off sensors for Fe^{3+} ions.* Analytical Methods, 2014. **6**(16): p. 6445-6451.
14. Tassist, A., et al., *Equilibrium, kinetic and thermodynamic studies on aluminum biosorption by a mycelial biomass (Streptomyces rimosus).* Journal of Hazardous Materials, 2010. **183**(1): p. 35-43.
15. Guan, X., et al., *Gallic acid-conjugated iron oxide nanocomposite: An efficient, separable, and reusable adsorbent for remediation of Al(III)-contaminated tannery wastewater.* Journal of Environmental Chemical Engineering, 2017. **5**(1): p. 479-487.
16. Lodeiro, P., et al., *Aluminium removal from wastewater by refused beach cast seaweed. Equilibrium and dynamic studies.* Journal of hazardous materials, 2010. **178**(1-3): p. 861-866.

17. Al-Muhtaseb, S.A., M.H. El-Naas, and S. Abdallah, *Removal of aluminum from aqueous solutions by adsorption on date-pit and BDH activated carbons.* Journal of Hazardous Materials, 2008. **158**(2-3): p. 300-307.
18. Andaç, M., et al., *Ion-selective imprinted beads for aluminum removal from aqueous solutions.* Industrial & engineering chemistry research, 2006. **45**(5): p. 1780-1786.
19. Islam, A., et al., *Selective separation of aluminum from biological and environmental samples using glyoxal-bis (2-hydroxyanil) functionalized Amberlite XAD-16 resin: kinetics and equilibrium studies.* Industrial & Engineering Chemistry Research, 2013. **52**(14): p. 5213-5220.
20. Aly, Z., et al., *Removal of aluminium from aqueous solutions using PAN-based adsorbents: characterisation, kinetics, equilibrium and thermodynamic studies.* Environmental Science and Pollution Research, 2014. **21**(5): p. 3972-3986.
